# Type 2-like polarization and elevated CXCL4 secretion of monocyte derived macrophages upon internalization of plasma-derived exosomes from head and neck cancer patients

**DOI:** 10.1186/s12885-024-12948-6

**Published:** 2024-09-20

**Authors:** Marie-Nicole Theodoraki, Diana Huber, Linda Hofmann, Lotte Werner, Christian Idel, Jonas Fleckner, Kirstin Plötze-Martin, Lutz Schütt, Cornelia Brunner, Reinhard Depping, Thomas K. Hoffmann, Karl-Ludwig Bruchhage, Ralph Pries

**Affiliations:** 1 Department of Otorhinolaryngology, Ulm University Medical Center, Ulm, Germany; 2 Department of Otorhinolaryngology, University of Luebeck, Luebeck, Germany; 3 Department of Otolaryngology, Head and Neck Surgery, School of Medicine and Health, Technical University of Munich (TUM), Munich, Germany; 4 Institute of Physiology, Working Group Hypoxia, University of Luebeck, Luebeck, Germany

**Keywords:** HNSCC, Macrophages, Exosomes, CD206, PD-L1, CTLA-4, CXCL4, Liquid biomarker

## Abstract

**Background:**

Exosomes are closely associated with different aspects of tumor-progression in patients with head and neck squamous cell carcinoma (HNSCC), such as angiogenesis or immune regulation. As extracellular vesicles they are involved in the intercellular communication by transferring their cargo such as proteins and nucleic acids from one cell to another. However, the influence of tumor related plasma-derived exosomes on the polarization and characteristics of monocyte derived macrophages is not fully understood.

**Methods:**

Exosomes were isolated from plasma samples of healthy donors (HD) and HNSCC patients and further evaluated with regard to morphology, size and protein composition via transmission electron microscopy, nanoparticle tracking, western blot analysis and cytokine assays. Differentiation and characteristics of monocyte derived macrophages upon exosome internalization were analyzed using flow cytometry and fluorescence microscopy. Macrophage cytokine secretion patterns were analyzed by human cytokine antibody arrays and ELISA measurements.

**Results:**

Our data revealed elevated overall plasma levels of CTLA-4, PD-L1, and TIM-3 as well as elevated exosome-associated CTLA-4, PD-L2, TIM-3, and LAG-3 levels in HNSCC patients compared to HD. Furthermore, we observed a significant type 2-like polarization and elevated CXCL4 secretion of monocyte derived macrophages upon internalization of plasma-derived exosomes from HNSCC patients, which could be visualized by fluorescence microcopy of membrane stained exosomes.

**Conclusions:**

The study provides new insights regarding exosome driven pro-tumorigenic immune regulation in the circulation of patients with head and neck cancer and could help to better understand the individual immunologic situation.

**Supplementary Information:**

The online version contains supplementary material available at 10.1186/s12885-024-12948-6.

## Introduction

Head and neck squamous cell carcinoma (HNSCC) is a widespread tumor entity originating primarily from the mucosal epithelium in the oral cavity, pharynx, and larynx [1, 2]. Available prognostic biomarkers such as histological tumor type, HPV (human papilloma virus) status or lymph node involvement are used to predict the individual situation and course of disease and are important for therapy decision making [3].

Development and progression of HNSCC are closely associated with cellular and molecular immunological alterations and immune escape mechanisms. In this context, immunotherapeutic targeting of different immune cells such as tumor infiltrating lymphocytes (TILs) and tumor associated macrophages (TAMs) is becoming increasingly important in clinical practice as a promising therapeutic addition [4–7].

Monocytes are macrophage precursors and together they constitute an important part of the mononuclear phagocyte system. Macrophages are classically subdivided into pro-inflammatory type 1 macrophages (M1) and anti-inflammatory type 2 (M2) macrophages. M1 macrophages are potent effector cells and a major source of pro-inflammatory cytokines such as Interleukin(IL)-1β, tumor necrosis factor (TNF)‐α, and IL‐6, while M2 macrophages attenuate Th1 immune responses and promote angiogenesis and tissue remodeling [8–10]. Both subtypes express the pan macrophage marker CD68, whereas M2 macrophages can be detected by the additional expression of scavenger receptor CD163 or mannose receptor CD206 [11, 12]. High expression of M2-polarized TAMs were associated with poor overall survival in oral squamous cell carcinoma (OSCC) patients [13], whereas it has recently been shown that especially CD206^+^ rather than CD163^+^ M2-like TAMs were the most abundant population within the tumor microenvironment (TME) in patients with laryngeal squamous cell carcinoma [14]. Tumor-associated M2-polarized macrophages are well known to be involved in different pro-tumorigenic aspects such as tumor growth and metastasis within the TME [15, 16]. However, the regulation and the involved communication pathways of macrophage differentiation in head and neck cancer are not yet fully understood.

Tumor-associated extracellular vesicles (EVs) are increasingly coming into focus with regard to the regulation of immune responses within the tumor microenvironment (TME).These small-sized (30–150 nm) vesicles are surrounded by a phospholipid bilayer and secreted by nearly all cells of the human body [17–20]. They are known to be involved in the systemic intercellular communication in the human body by delivering their cargo including proteins and nucleic acids from one cell to another [21, 22], whereas their cellular interaction and uptake is mediated by surface exposed proteins. Exosomes from different types of cancer are associated with tumor-promoting effects, angiogenesis, or immune alterations, respectively [18]. Furthermore, checkpoint molecule PD-L1 (programmed death ligand 1; CD274) has been detected on the surface of plasma derived exosomes in HNSCC patients in correlation with the individual tumor- and lymph node status [23].

Plasma samples of HNSCC patients with advanced stage of disease have shown a significantly higher proportion of plasma derived CD16^+^ exosomes, which is furthermore associated the individual distribution of circulating monocyte subsets [24, 25]. TAMs polarize into the M2 phenotype and suppress anticancer immune responses by secretion of cytokines such as IL-4, IL‐13, and IL‐10, Toll‐like receptor ligands, and glucocorticoids [8, 26]. However, the influence of plasma derived HNSCC exosomes with regard to the M1/M2 macrophage polarization patterns and the immunological characteristics have not been investigated in detail.

Therefore, using the human monocyte leukemia cell line THP-1 (Tohoku Hospital Pediatrics-1) [27] as a model, we investigated the influence of plasma-derived exosomes from HNSCC patients and healthy donors on the M1/M2 differentiation of human macrophages and the expression patterns of checkpoint molecules PD-L1 and CTLA-4 (cytotoxic T-lymphocyte-associated protein 4; CD152). Moreover, exosomal levels of checkpoint molecules PD-L1, PD-L2, CTLA-4, TIM-3 (T cell immunoglobulin and mucin-domain containing-3), and LAG-3 (Lymphocyte activation gene 3 protein) were analyzed. Furthermore, comprehensive evaluation of cytokine secretion patterns in response to exosome internalization was carried out in correlation with the immunological situation.

This study aimed to broaden our understanding of the interplay of TAMs and plasma exosomes as bioliquid immune regulators in patients with head and neck cancer.

## Results

### Evaluation of plasma derived exosomes

**Fig. 1:**
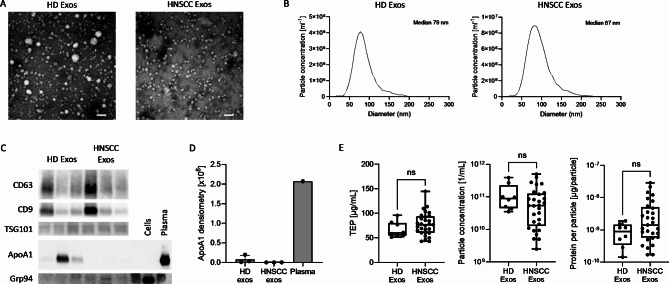
Characterization of plasma exosomes. (**A**) Representative transmission electron microscopy images and (**B**) size distribution profiles of exosomes from plasma of a healthy donor (HD) and a patient with head and neck squamous cell carcinoma (HNSCC). Scale bar = 100 nm. (**C**) Representative western blot images from plasma-derived exosomes. (**D**) Quantification of ApoA1 band intensity from (**C**). (**E**) Comparison of total exosome protein (TEP), particle concentration and protein per particle between *n* = 8 HD and *n* = 29 HNSCC patients. Box-and-whiskers show median, 25th and 75th quartiles and range. ns = not significant, as determined by Mann-Whitney test

Plasma derived exosomes isolated from plasma of healthy donors (HD) and HNSCC patients were evaluated for morphology, size and protein composition by transmission electron microscopy, nanoparticle tracking and western blot analysis. Data revealed a circular shape (Fig. 1A) and ranged from 30 to 200 nm with median diameters around 90 nm (Fig. 1B). western hybridization experiments revealed positivity the tetraspanins CD63 and CD9 and the endosomal marker TSG101 and were shown to be mostly negative for the non-exosomal proteins Grp94 and apolipoprotein ApoA1 (Fig. 1C, D). Non-significant differences were seen in total exosome protein (TEP) levels, particle concentration, or protein per particle (Fig. 1E). Data are in accordance with the MISEV 2018 guidelines for exosome nomenclature [28].

### Surface levels of immune-checkpoints on exosomes

**Fig. 2:**
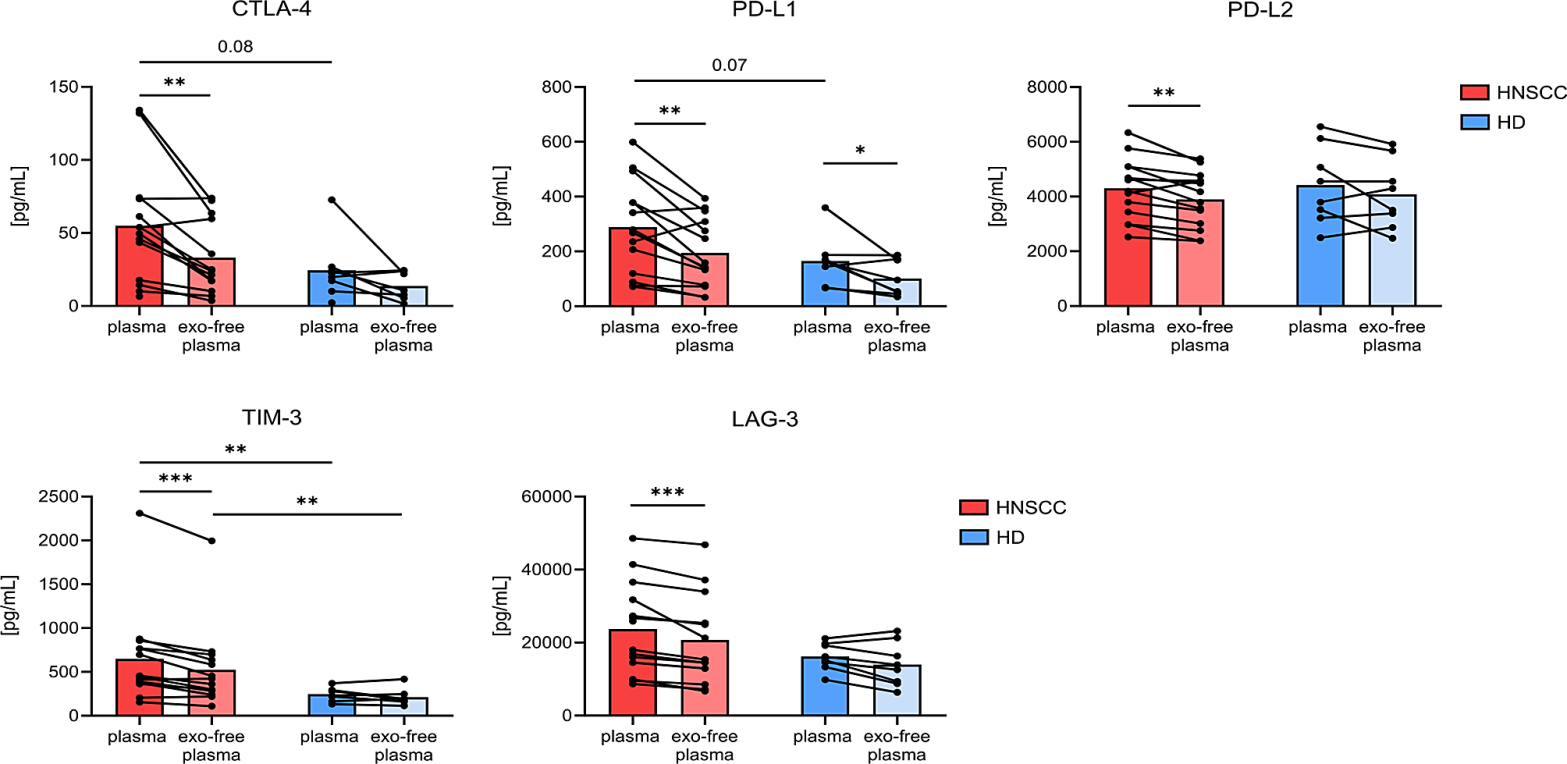
Comparison of immune modulators in plasma before and after depletion of exosomes. Levels of CTLA-4, PD-L1, PD-L2, TIM-3 and LAG-3 were measured with Milliplex Multiplex Assay in plasma of HNSCC patients (*n* = 14) and HD (*n* = 8) before (plasma) and after (exo-free plasma) depletion of exosomes by ultracentrifugation. Differences in plasma before and after exosome depletion were compared by paired Wilcoxon test, differences between HNSCC patients and HD by Mann-Whitney test

Next, indirect assessment of exosomal surface levels of immune-checkpoint molecules PD-L1, PD-L2, CTLA-4, TIM-3, and LAG-3 was performed by measuring plasma versus exosome-depleted plasma of HD and HNSCC patients using multiplex bead-based immunoassays. Our data revealed elevated overall plasma levels of CTLA-4, PD-L1, and TIM-3 in HNSCC compared to HD. However, significant differences were only seen for TIM-3. Besides PD-L1, where a significant reduction between plasma and exosome-depleted plasma was visible in HNSCC patients *and* in HD, for all other checkpoint molecules this observation was *only* made in HNSCC patients, indicating elevated surface levels in HNSCC plasma-derived exosomes (Fig. 2).

### Macrophage polarization upon exosome internalization

**Fig. 3:**
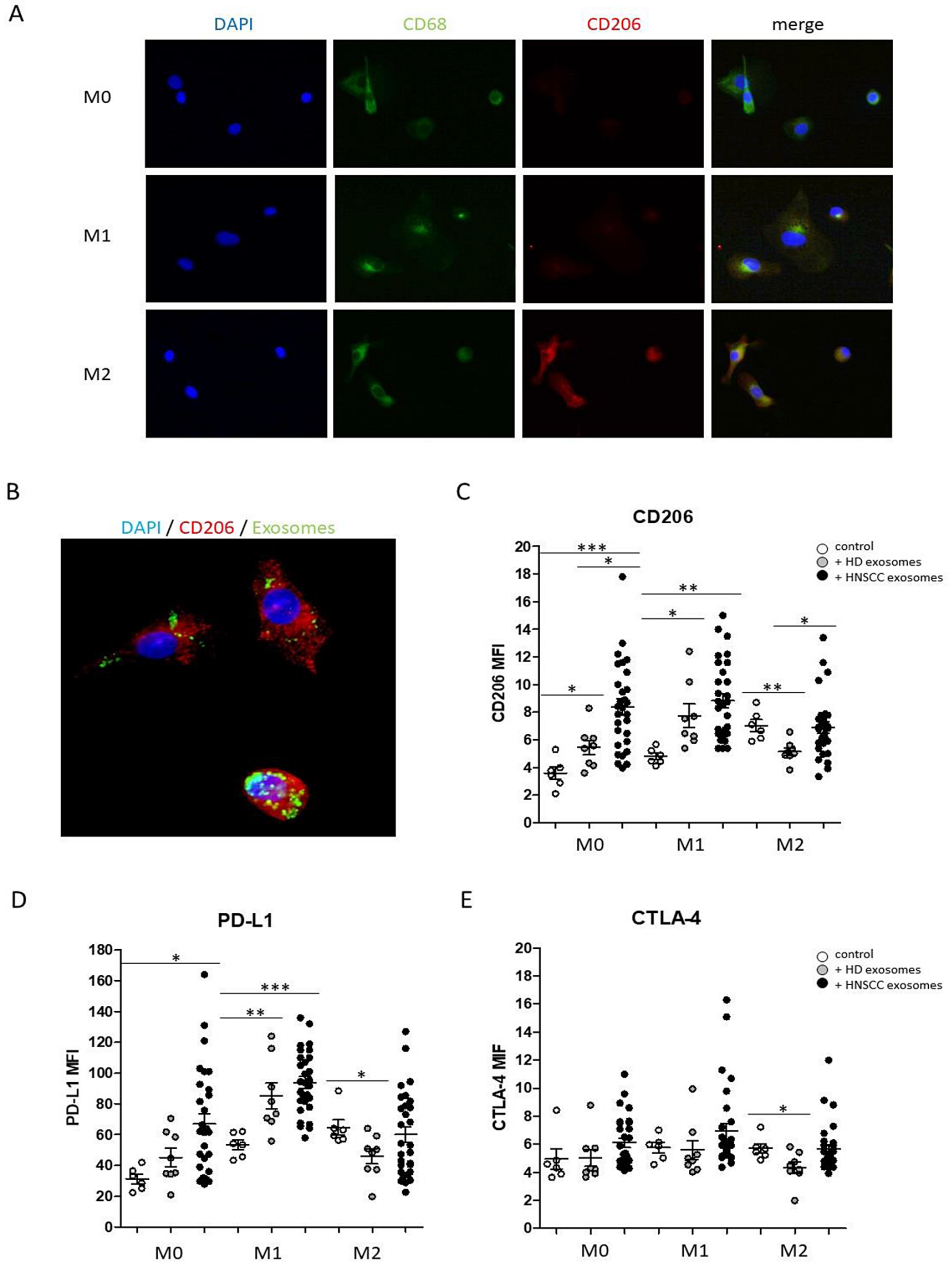
Macrophage polarization of THP-1 monocytes in response to plasma derived exosomes from HDs and HNSCC patients, compared to the control (absence of exosomes). (**A**) Polarization of THP-1 monocytes into type 1-like macrophages (induced by 20 ng/ml IFN, 10 pg/ml LPS) and type 2-like macrophages (induced by 20 ng/ml IL-4, 20 ng/ml IL-13) compared to non-polarized (M0) macrophages via immunohistochemical staining of M2-like macrophage marker CD206 and pan macrophage marker CD68. Blue fluorescence shows DAPI stained nuclei, green fluorescence shows FITC-anti-CD68 staining and red fluorescence shows dsRed-anti-CD206 staining. (**B**) Representative exosomal internalization by monocyte derived macrophages was visualized by FITC-fluorescence staining of the exosomal membrane (green). Blue fluorescence shows DAPI stained nuclei and red fluorescence shows dsRed-anti-CD206 staining. (**C-E**) Expression of M2-like macrophage marker CD206 (**C**), immune checkpoint molecule PD-L1 (**D**) and CTLA-4 (**D**) under M0 and M1 and M2 culturing conditions in response to both HD and HNSCC exosomes. *: *p* < 0.05; **: *p* < 0.01; ***: *p* < 0.001. MFI: mean fluorescence intensity

In vitro polarization of THP-1 monocytes into type 1-like macrophages (induced by 20 ng/ml IFN, 10 pg/ml LPS) and type 2-like macrophages (induced by 20 ng/ml IL-4, 20 ng/ml IL-13) compared to non-polarized (M0) macrophages was visualized via immunohistochemical staining of M2-like macrophage marker CD206 and pan macrophage marker CD68, which does not differentiate between macrophage subtypes (Fig. 3A).

Next, polarization of THP-1 monocytes into M0/M1/M2 macrophages was performed in the absence (control) or presence of 10 µg/ml plasma-derived exosomes from HNSCC patients or HD, whereas fluorescence staining of the exosomal membrane revealed an efficient cellular uptake (Fig. 3B) with no obvious differences between the distinct differentiation conditions (data not shown).

Next, expression patterns of M2-like macrophage marker CD206 and checkpoint molecules PD-L1 and CTLA-4 on monocyte derived macrophages in response to exosome internalization was analyzed using flow cytometry.

Data revealed significantly increased expression patterns of CD206 under M0 and M1 culturing conditions in response to both HD and HNSCC exosomes, with significantly stronger effects of exosomes from HNSCC patients. No increased CD206 was observed in M2-like macrophages in response to HNSCC exosomes, but CD206 expression was found to be significantly reduced in M2 polarized macrophages in response to exosomes from HD (Fig. 3C). Data revealed significantly increased expression levels of checkpoint molecule PD-L1 under M0 culturing conditions in response to HNSCC exosomes and significantly increased PD-L1 expression under M1 culturing conditions in response to both HD and HNSCC exosomes, with significantly stronger effects of exosomes from HNSCC patients (Fig. 3D). In contrast, CTLA-4 expression was not significantly increased in response to exosome internalization (Fig. 3E). Surprisingly, expression levels of checkpoint molecules PD-L1 and CTLA-4 were as well found to be significantly decreased in M2 polarized macrophages in response to exosomes from HD (Fig. 3D, E).

### Cytokine secretion upon exosome internalization

**Fig. 4:**
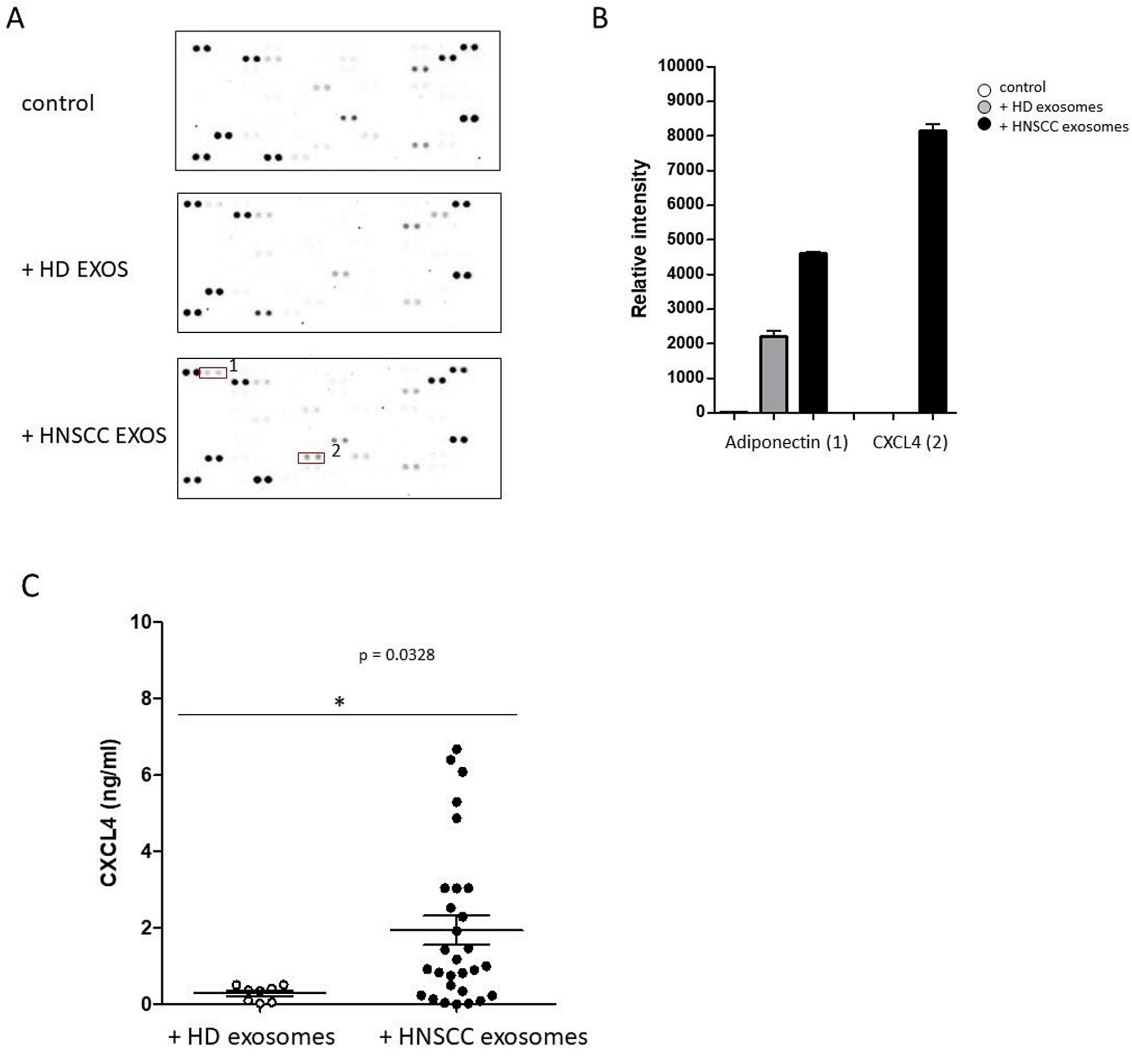
Cytokine screening upon internalization of plasma derived exosomes. (**A**) Raw images of cytokine arrays of culture supernatants from monocyte derived macrophages upon internalization of plasma derived exosomes from HDs and HNSCC patients. Numbers indicate increased densities of bands of certain cytokines in response to HNSCC exosomes (1: Adiponectin; 2: CXCL4). (**B**) Semiquantitative analysis was performed by measuring the density of the dots and revealed differential secretion patterns of different cytokines. (**C**) Quantification of CXCL4 supernatant concentrations (ng/ml) by ELISA measurements revealed significantly increased concentrations of chemokine CXCL4 in response to plasma exosomes from HNSCC patients compared to HDs. *: *p* < 0.05

To determine cytokine secretion patterns of THP-1 monocytes in responses to the internalization of plasma derived exosomes from HNSCC patients and HDs, expression levels of 105 different cytokines and chemokines in supernatants of treated monocyte cell cultures were screened using a human cytokine antibody array (Fig. 4A).

Semiquantitative analyses were performed by measuring the density of the dots and revealed increased secretion levels of adiponectin and CXCL4 in response to HNSCC exosomes (Fig. 4B). Furthermore, quantification of CXCL4 concentrations of all supernatants by ELISA measurements revealed significantly increased concentrations of chemokine CXCL4 in response to plasma exosomes from HNSCC patients compared to exosomes from HDs (Fig. 4C).

**Fig. 5:**
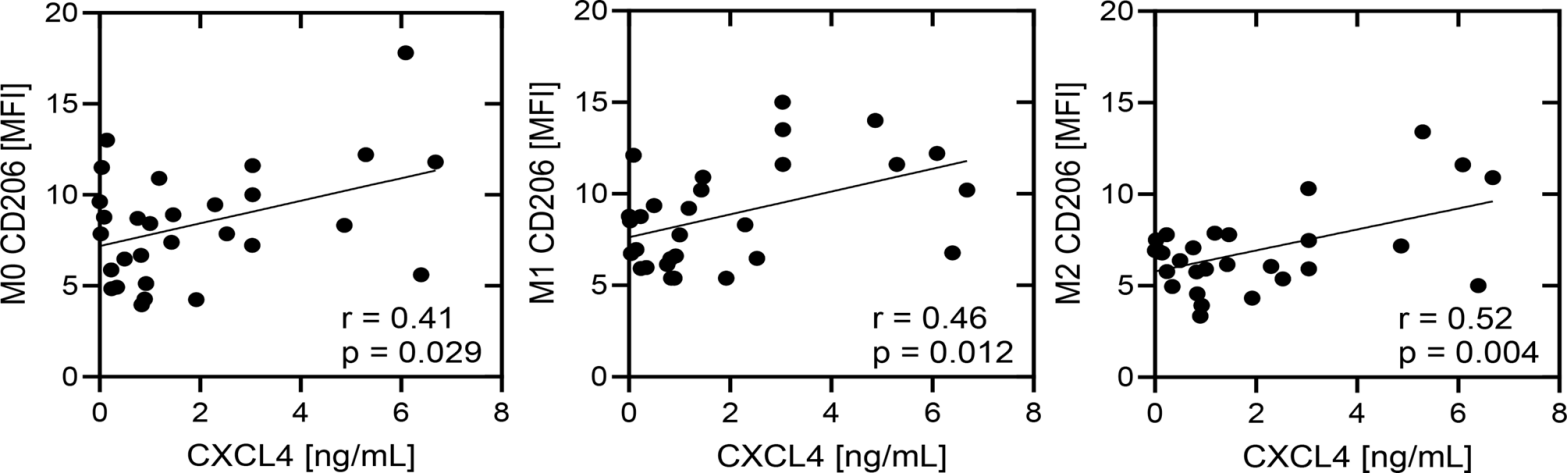
Correlation analysis between secreted CXCL4 (ng/ml) and expression levels of M2-like macrophage marker CD206 on monocyte derived M0/M1/M2 macrophages upon HNSCC exosome internalization. The correlation coefficient (r) and p values are given for each correlation. *p* < 0.05 was considered as significant. MFI: mean fluorescence intensity

In order to further elucidate the potential connection between the induced CXCL4 secretion and the M2 polarizing impact of the corresponding exosomes of our HNSCC patient cohort, correlation analyses between measured CXCL4 (ng/ml) and expression levels of M2-like macrophage marker CD206 on monocyte derived M0/M1/M2 macrophages upon HNSCC exosome internalization were performed. Our data revealed significant positive correlations in all analyzed macrophage subtypes, which underlines the systemic association between M2 polarization and CXCL4 secretion in response to plasma exosomes in patients with head and neck cancer (Fig. 5).

Further correlation analyses between CXCL4 concentrations and characteristics of plasma derived exosomes from HNSCC patients and HDs were performed.

**Fig. 6:**
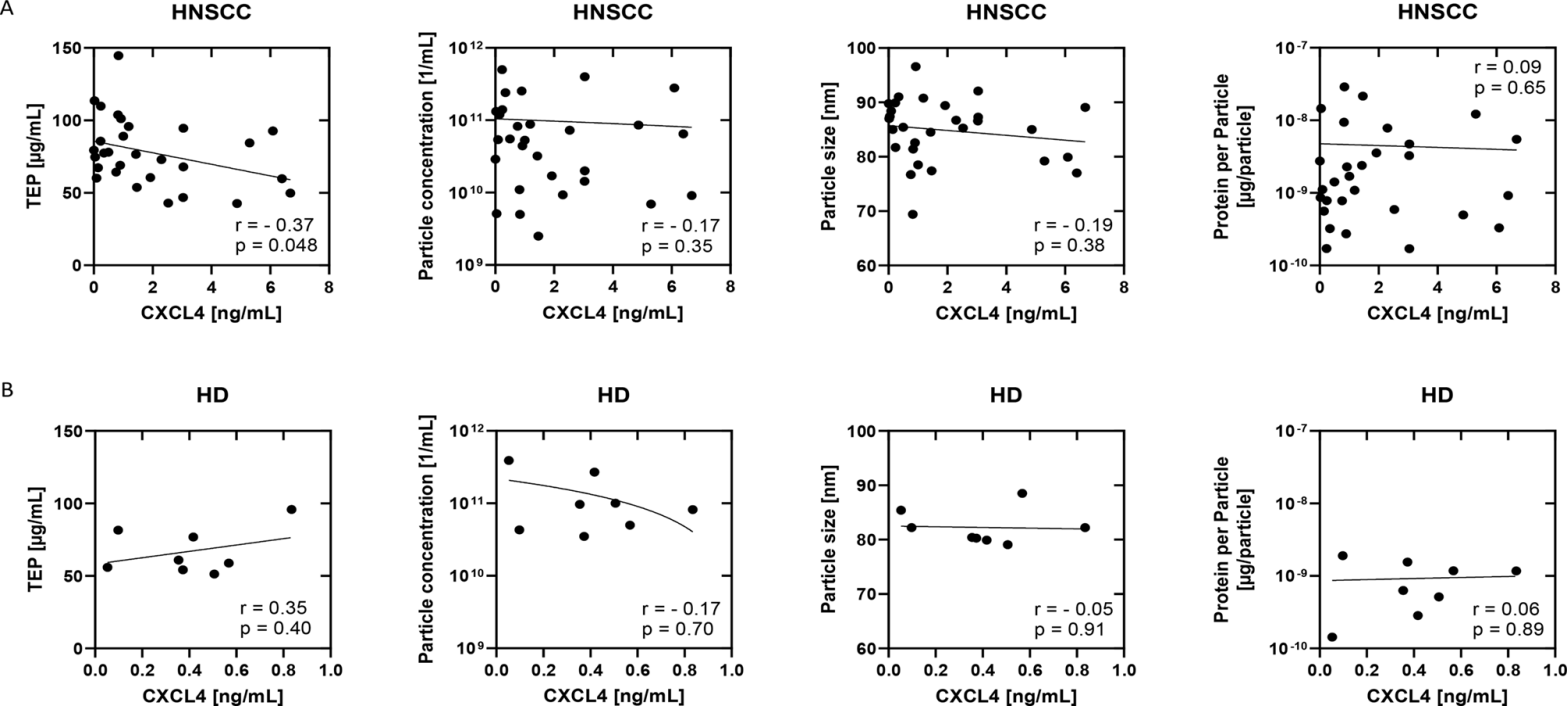
Correlation of exosome characteristics and CXCL4 in HNSCC patients. Scatter plots of correlation analysis of (**A**) *n* = 29 HNSCC and (**B**) *n* = 8 HD between CXCL4 levels and TEP, particle concentration, particle size or protein per particle. Dependent on the data distribution, Spearman or Pearson correlation analysis was applied. *p* < 0.05 was considered as significant

Our data revealed a significant negative correlation between CXCL4 (ng/ml) concentrations and TEP in HNSCC patients but not in HDs (Fig. 6). Data revealed no significant correlation between CXCL4 levels and values for tumor site, stage, or HPV status (data not shown).

## Materials and methods

### Ethics statement

Patients of the present study were examined and treated at the Department of Otorhinolaryngology, University Hospital Schleswig-Holstein, Campus Luebeck. Inclusion criteria were patients with age greater than 18 years and a pathologically confirmed HNSCC subsites of oropharynx, larynx, oral cavity, nasopharynx, paranasal sinuses, and hypopharynx.

We included patients with p16 positive and negative HNSCC. Exclusion criteria were patients with incomplete treatment records and unclear histology. The study was conducted after written informed consent of the participating patients and approved by the ethics committee of the University of Luebeck (approval number 16–278).

### Blood samples and clinicopathological data

Blood samples were drawn into sodium citrate containing S-Monovettes (Sarstedt; Nümbrecht, Germany) from HDs (*n* = 8; mean age of 59) and HNSCC patients (*n* = 29; mean age of 64). After centrifugation at 1000× *g* for 10 min, obtained plasma samples were stored at − 80 °C. Clinicopathological parameters are listed in Table 1.

**Table 1:** Clinicopathological parameters

Characteristics	Patients (*n* = 29)	
	*n*	%
**Gender**		
M	18	62
F	11	38
**Tumor site**		
Pharynx	15	51
Larynx	5	17
Oral cavity	9	32
**Tumor stage**		
T1 - T2	13	64
T3 -T4	16	36
**Lymph node metastasis**		
N (O)	7	24
N (+)	22	76
**Distant metastasis**		
M (0)	21	72
M (+)	8	28
**HPV status**		
Positive	11	38
Negative	11	38
Unknown	7	24
**Alcohol abuse**		
Yes	5	17
No	24	83
**Tobacco consumption**		
Yes	21	72
No	8	28

### Exosome isolation

Size exclusion chromatography was used to isolated exosomes from plasma samples as described before [29]. Briefly summarized, plasma samples were sequentially centrifuged (2000× *g* for 10 min and 12000× *g* for 30 min at 4 °C) and supernatants were filtered (0.22 μm syringe-driven filters; Millipore, Burlington, MA, USA) and then loaded on pre-packed sepharose columns. Concentrations of total exosome protein were measured using Pierce BCA Protein Assay (Thermofisher Scientific, Waltham, MA, USA).

### Characterization of exosomes

Western Blot was used to confirm the cellular origin of the vesicles by detecting the endosomal marker TSG101, and the exosome-associated tetraspanins CD9 and CD63. Transmission electron microscopy (TEM) was used to analyze the morphology of the isolated vesicles, and nanoparticle tracking analysis (NTA) for size and particle counting. These methods are in accordance with the minimal information for studies of extracellular vesicle (MISEV) 2018 guidelines [28] and are routinely performed as described in our previous publication [24] (EV-TRACK ID: EV200068).

### Immune checkpoint detection in plasma and exosome-depleted plasma by luminex multiplex assay

Levels of cytokines TIM3, LAG-3, CTLA-4, PD-L1 and PD-L2 were measured in plasma samples from HNSCC patients and HDs. To investigate the effect of exosomes indirectly, exosome-depleted plasma samples were measured, prepared by ultracentrifugation at 110,000 xg for 2 h. Samples were analyzed using the Milliplex Multiplex Assay (HCKP1-11 K-05) from Merck Millipore according to the manufacturer’s instructions. Briefly, a 96-well plate was primed with washing buffer, samples or standard were added to each well together with assay buffer and magnetic beads and incubated at 4 °C overnight. Afterwards, the beads were washed with washing buffer using a magnetic plate to discard the liquid. Detection antibodies were added for 1 h followed by streptavidin-phycoerythrin for another 30 min. After washing again, the beads were resuspended in Sheath Fluid and the plate was measured by Luminex 200 with Luminex xMAP technology and median fluorescent intensities (MFI) of the analytes were used for analysis.

### THP-1 culture conditions and macrophage polarization

For cell culture experiments the non-adherent monocyte cell line THP-1 was used. Cell culture was performed in RPMI 1640 medium supplemented with 10% heat inactivated fetal bovine serum (FBS), 1% sodium pyruvate and 1% streptomycin/penicillin at 37 °C and 5% CO_2_ under a humidified atmosphere. Cells were subcultured every 3 days when they reached a maximum density of 1 × 10^6^ cells/ml. To induce differentiation into monocyte-derived macrophages (MDMs), THP-1 cells were treated with 5 ng/ml phorbol-12-myristate-13-acetate (PMA) for 24 h, before cells were further cultivated in fresh medium. After a four-day resting period, cells became adherent and developed a macrophage like morphology. Next, MDMs were further differentiated into type 1-like macrophages (20 ng/ml IFN, 10 pg/ml LPS) and type 2-like macrophages (20 ng/ml IL-4, 20 ng/ml IL-13) in the absence or presence of 10 µg/ml plasma derived exosomes from HNSCC patients or HDs for 48 h. To generate non-polarized (M0) macrophages, THP-1 monocytes were differentiated with PMA for 24 h and cultured further in standard medium. The cells were then washed with PBS and used for FACS analysis.

### Staining of THP-1 cells and FACS analysis

Flow cytometry was performed with a MACSQuant 10 flow cytometer (Miltenyi Biotec, Bergisch-Gladbach, Germany). Therefore, cells were stained for 25 min in the dark the with following antibodies: CD152-PE-Cy7, CD206-PerCP, and PD-L1-APC (all from Biolegend, San Diego, USA). Subsequently, 700 µl RBC Lysis Buffer (Biolegend) were added for another 20 min and suspension was centrifuged at 400 x g for 5 min and supernatant was discarded. Cell pellet was resuspended in 100 µl fresh PBS and measured using flow cytometry. Obtained data were evaluated with the FlowJo software version 10.0 (FlowJo, LLC, Ashland, USA).

### Cytokine analyses

Cytokine expression patterns of THP-1 monocytes in response the internalization of isolated exosomes from HNSCC patients and HDs were analyzed using Proteome Profiler^™^ Human XL cytokine arrays (R&D Systems, Minneapolis, MN, USA) as recommended by the supplier. Therefore, supernatants from cell cultures were collected after incubation and instantly frozen with liquid nitrogen and preserved at -80 °C. Membranes were hybridized with the cell culture medium and cytokine expression was visualized using an enhanced chemiluminescence detection kit (R&D Systems, Minneapolis, MN, USA). Semiquantitative analysis was performed by measuring the density of the bands using an iBright CL 1000 biomolecular imager (Invitrogen, Carlsbad, CA, USA). Secretion levels of chemokine CXCL4 were determined via ELISA assays (R&D Systems, Minneapolis, MN, USA).

### Exosome internalization

THP-1 monocytes (2.10^5^ cells/well) were seeded on 4-well-chambered slides together with 1 ml RPMI differentiation medium and 2.5 µl PMA per ml. After 24 h, the adherent cells got a medium change and 72 h later, differentiation medium with 250ng/ml LPS and 20ng/ml IFNγ was added to the cells for differentiation into M1 macrophages or differentiation medium with 20ng/ml IL4 and 20ng/ml IL13 for differentiation into M2 macrophages. Isolated exosomes were labeled with PKH67 membrane-labeling solution (Sigma-Aldrich, #PKH67GL-1KT) according to the manufacturer’s instructions. Briefly, 10 µg exosomes per batch were diluted and incubated with 2 µL PKH dye for 10 min at RT in the dark to stain the exosomal membrane. Excessive dye was removed by centrifugation. After 48 h, macrophages were fixed using ice-cold methanol acetone and analyzed by fluorescence microscopy.

### Statistical analyses

Statistical analyses were carried out using GraphPad Prism Version 7.0f (GraphPad Software, Inc., San Diego, CA, USA) and pairwise comparison of data was performed using student´s t-Tests or non-parametric Mann-Whitney test. Correlations were calculated using multivariate regression with the Pearson correlation coefficient. *p* < 0.05 (*), *p* < 0.01 (**), and *p* < 0.001 (***). Additional statistical details are given in the respective figure legends, when appropriate.

## Discussion

The HNSCC microenvironment consists of innumerable soluble mediators such as cytokines and growth factors that contribute to tumor progression and immune evasion [30–32], whereas the role of exosomes within these intercellular communication pathways is less well understood. Therefore, the present study was undertaken to investigate the influence of plasma-derived exosomes from HNSCC patients on the differentiation and immune regulation of monocyte-derived macrophages. Plasma exosomes have been identified as promising bioliquid indicators for treatment response and tumor progression in head and neck cancer [33]. In this context, it has been shown that exosomes contribute to the HNSCC immunosuppressive microenvironment via elevated levels of checkpoint molecule PD-L1 [23], whereas further investigations revealed distinct cellular origins of PD-L1-positive extracellular vesicles in HNSCC patients, including tumor cells, T cells, and B cells, but also monocytes and macrophages [34].

By an indirect assessment of exosomal immune-checkpoint surface levels our data revealed significantly reduced expression levels of all measured checkpoint molecules (PD-L1, PD-L2, CTLA-4, TIM-3, LAG-3) between plasma and exosome-free plasma. The difference in signal between plasma and exo-free plasma is assumed to derive from exosomes. We observed elevated overall plasma levels of CTLA-4, PD-L1, TIM-3 in HNSCC compared to HD, which is not surprising, since all measured immune checkpoints are highly immunosuppressive.

Significant reduction of all 5 markers between plasma and exo-free plasma was visible only in HNSCC patients and not in HD, indicating elevated surface levels in HNSCC plasma-derived exosomes compared to HD. We and others have shown before, that plasma-derived exosomes from cancer patients -especially HNSCC- have a highly immunosuppressive cargo, which can be used for modulation of recipient immune cell functions [23, 35]. Additionally to our previous findings we demonstrate here not only elevated levels of PD-L1, PD-L2 and CTLA-4, but also TIM-3 and LAG-3, both immune checkpoint molecules known to promote immunosuppression in the TME [36, 37].

Furthermore, our data revealed an increased type 2-like shifted differentiation of monocyte derived macrophages upon internalization of plasma derived exosomes from HNSCC patients compared to exosomes from HDs. However, also internalization of exosomes from HDs resulted in increased expression levels of M2-like marker CD206 under M0 and M1 culturing conditions. These data suggest a general exosome-associated activation of the macrophage phagocytic signaling and certain M2 like characteristics, as it has recently been proposed in a study on myocardial ischemic injury [38].

In contrast, our data revealed reduced expression levels of CD206, PD-L1 and CTLA-4 in M2-like macrophages in response to plasma derived exosomes from HDs, suggesting a partial M1 shift in this situation. Similarly, it has been reported that exosomes from M1-polarized macrophages potentiate cancer vaccines by creating a pro-inflammatory microenvironment in melanoma patients [39].

M2 like tumor associated macrophages are known to be pro-tumorigenic and to be correlated with worse survival of HNSCC patients [40]. Recently, it has been shown that oral cancer stem cell-derived small extracellular vesicles are able to promote type 2-like macrophage polarization via transferring specific long non-coding RNAs (lncRNA) [41]. Similarly, it was suggested that exosomal lncRNA from laryngeal carcinoma tissues were capable to induce type 2-like macrophage polarization via the PI3K/p-AKT/AKT signaling pathway [42] and also exosomes from pancreatic cancer cell lines increased the expression patterns of markers indicative for a an immunosuppressive M2-like macrophage polarization [43]. These data underline a significant impact of small extracellular vesicles from different cell types, tissues and body fluids on the differentiation patterns of tumor associated macrophages (TAMs). TAMs within the tumor microenvironment predominantly reveal M2-like properties and promote tumor growth and progression, whereas M1- like TAMs are supposed to be correlated with better survival [15, 16, 40].

We identified a significant upregulation of checkpoint molecules PD-L1 and CTLA-4 on monocyte derived macrophages upon internalization of HNSCC plasma exosomes, both of which are the two most representative immune checkpoint pathways and are well known to negatively regulate anti-tumor T cell immune functions.

Basically, it is common knowledge, that proinflammatory cytokines such as interferon (IFN)-γ, interleukin (IL)-1β, or tumor necrosis factor (TNF)-α act as inducers of typ-1 like macrophage polarization, whereas ‘alternative M2 macrophages are induced in response to IL-4 and IL-13. M2 macrophages are known to attenuate the inflammatory process and to promote angiogenesis and tissue repair [44, 45].

Our data revealed significantly increased secretion patterns of adiponectin and of chemokine CXCL4 (platelet factor 4) by THP1 monocytes in response to plasma derived exosomes from HNSCC patients compared to HDs. Adiponectin acts as a key regulator of the innate immune system and has been shown to prime human monocytes into alternative M2 macrophages. Moreover, adiponectin-treated monocytes revealed increased M2 markers, such as mannose receptor (MR) and alternative macrophage activation-associated CC chemokine-1. Furthermore, incubation of M1 macrophages with supernatants of adiponectin-treated M2-derived macrophages resulted in an inhibition of tumor necrosis factor-α and monocyte chemotactic protein-1 secretion [46, 47].

Chemokine CXCL4 is mainly produced by activated platelets, but certain somatic cells, leukocytes and cancer cells also express CXCL4. However, the immunological function of non-platelet-derived CXCL4 is unclear. CXCL4 has been shown to promote monocyte survival and the binding of monocytes to the endothelial wall of the blood vessels, what supports their extravasation into the tissue [48, 49].

It has recently been shown that hypoxia is fundamental for CXCL4 production by umbilical cord CD34 derived plasmacytoid dendritic cells, via an overproduction of mitochondrial reactive oxygen species (mtROS) and the stabilization of HIF-2α [50]. Increased CXCL4 secretion levels from THP-1 monocytes were as well observed in response to 5% hypoxia growth conditions [51]. Furthermore, it has been shown that CXCL4 induce differentiation of monocytes into myeloid-derived suppressor cells (MDSCs), which inhibits CD8^+^ T-cell function and thus supports cancer metastasis [52]. Moreover, colon cancer studies discovered, that CXCL4 secreted by cancer cells accelerated tumor growth by inhibiting the antitumor activities of cytotoxic T lymphocytes (CTLs) [53]. Transcriptome analyses revealed that CXCL4 induced macrophages overexpressed certain M1 and M2 markers, which was not entirely consistent with neither M1 nor M2 transcriptomes [54], which underlines the need for further investigations.

An interesting observation was the negative correlation between exosome induced monocytic CXCL4 secretion and total exosome protein levels only in HNSCC patients and not in HD. In previous (unpublished) results from our group we have shown that the main way of exosome-dependent modulation of the recipient cell protein cargo is not a direct protein transfer but a transfer of exosomal miRNAs, which modulate the transcription in the recipient cell [55, 56]. A reduction in TEP may be caused by an increase in miRNAs in the exosomal lumen, which are responsible for the CXCL4 increase in macrophages. Further investigations on macrophage cytokine secretion levels in response to exosome uptake may help to identify novel relevant biomarkers to better understand the underlying regulatory network.

It is well known that extracellular vesicles released by tumor cells participate in the communication between tumor cells and immune cells, either in the tumor microenvironment or in the circulation [57]. It has been shown that circulating extracellular vesicles from rectal cancer patients induce the secretion of different cytokines after internalization by monocytes. Even different transcriptional changes were found in monocytes receiving EVs from patients with metastatic compared with non-metastatic cancer [58], impliyng a predictive value of monoycte-derived cytokines to monitor metastasis occurence. Further comprehensive investigations on larger patient cohorts in correlation with the specific individual therapy regimen and therapy response will help to further elucidate the meaningfulness of plasma-derived exosomes and chemokine CXCL4 as potential bioliquid indicators in head and neck cancer.

## Electronic supplementary material

Below is the link to the electronic supplementary material.

Supplementary Material 1

## Data Availability

Data is provided within the manuscript or supplementary information files.
