# Supplementary figures and images for: Type 2-like polarization and elevated CXCL4 secretion of monocyte derived macrophages upon internalization of plasma-derived exosomes from head and neck cancer patients
Source: BMC Cancer. 2024 Sep 20;24:1173. doi: 10.1186/s12885-024-12948-6 (PMC11414076; doi:10.1186/s12885-024-12948-6)

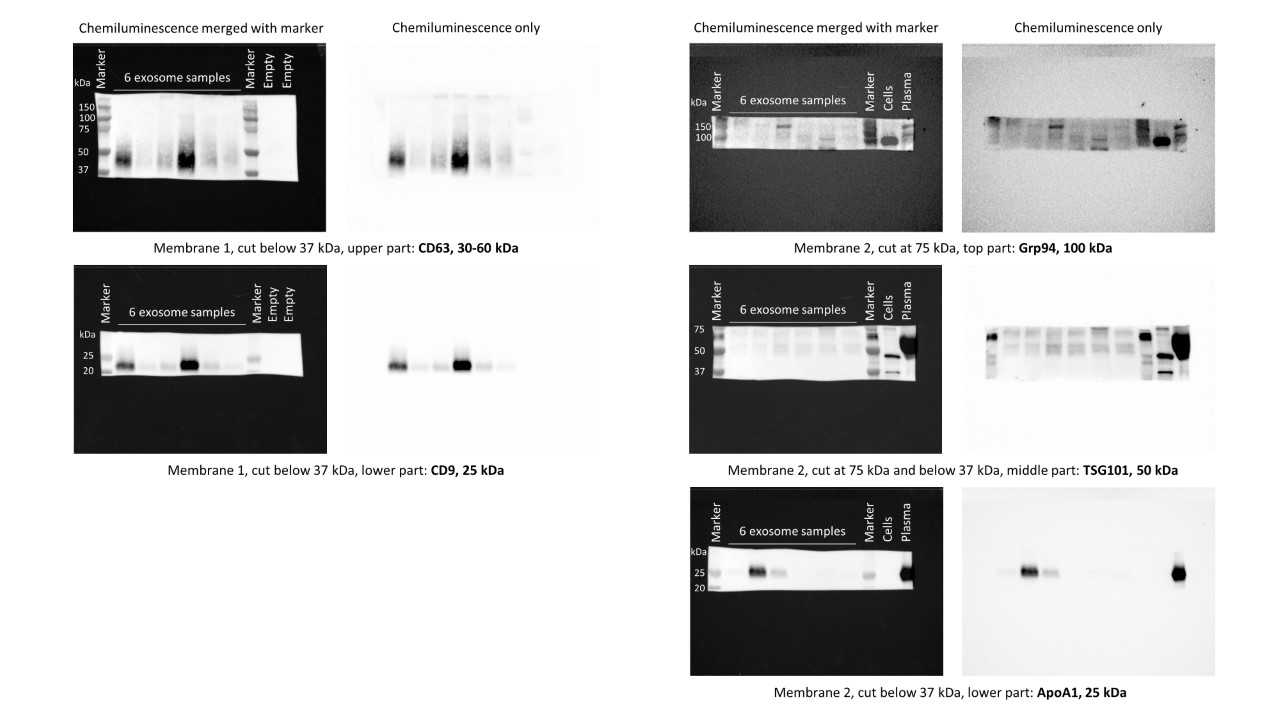

Supplement: Supplementary file 1 — Supplementary Material 1 [file 12885_2024_12948_MOESM1_ESM.jfif]
